# Non-Syndromic Intellectual Disability and Its Pathways: A Long Noncoding RNA Perspective

**DOI:** 10.3390/ncrna7010022

**Published:** 2021-03-11

**Authors:** Isabela I. Barros, Vitor Leão, Jessica O. Santis, Reginaldo C. A. Rosa, Danielle B. Brotto, Camila B. Storti, Ádamo D. D. Siena, Greice A. Molfetta, Wilson A. Silva

**Affiliations:** 1 Department of Genetics at the Ribeirão Preto Medical School, University of São Paulo, Avenida Bandeirantes 3900, Monte Alegre, Ribeirão Preto, São Paulo 14049-900, Brazil; isabelaib@usp.br (I.I.B.); leao.vitorleao@alumni.usp.br (V.L.); jessica.santis@usp.br (J.O.S.); reginaldoallves@usp.br (R.C.A.R.); daniellebrotto@alumni.usp.br (D.B.B.); camilabaldin.s@usp.br (C.B.S.); adamo@usp.br (Á.D.D.S.); gamolf@fmrp.usp.br (G.A.M.); 2 National Institute of Science and Technology in Stem Cell and Cell Therapy and Center for Cell Based Therapy, Ribeirão Preto Medical School, University of São Paulo, Rua Tenente Catão Roxo, 2501, Monte Alegre, Ribeirão Preto 14051-140, Brazil; 3 Center for Integrative Systems Biology-CISBi, NAP/USP, Ribeirão Preto Medical School, University of São Paulo, Rua Catão Roxo, 2501, Monte Alegre, Ribeirão Preto 14051-140, Brazil; 4 Department of Medicine at the Midwest State University of Paraná-UNICENTRO, and Guarapuava Institute for Cancer Research, Rua Fortim Atalaia, 1900, Cidade dos Lagos, Guarapuava 85100-000, Brazil

**Keywords:** long noncoding RNA, non-syndromic intellectual disability, molecular and cellular pathways, neurodevelopment, neural function

## Abstract

Non-syndromic intellectual disability (NS-ID or idiopathic) is a complex neurodevelopmental disorder that represents a global health issue. Although many efforts have been made to characterize it and distinguish it from syndromic intellectual disability (S-ID), the highly heterogeneous aspect of this disorder makes it difficult to understand its etiology. Long noncoding RNAs (lncRNAs) comprise a large group of transcripts that can act through various mechanisms and be involved in important neurodevelopmental processes. In this sense, comprehending the roles they play in this intricate context is a valuable way of getting new insights about how NS-ID can arise and develop. In this review, we attempt to bring together knowledge available in the literature about lncRNAs involved with molecular and cellular pathways already described in intellectual disability and neural function, to better understand their relevance in NS-ID and the regulatory complexity of this disorder.

## 1. Intellectual Disability

Intellectual disability (ID) is characterized by significant intellectual functioning limitation and adaptive behavior occurring before the age of 18 [1]. It is usually defined by an intelligence quotient (IQ) of less than 70 and severe deficiency in the environment and social milieu adaptation [2], which can be caused by genetic, prenatal, and environmental factors, representing a health issue in both developed and developing countries [3].

ID can be classified by severity into five categories (mild, moderate, severe, profound, and unable to classify), and it is also divided into syndromic intellectual disability (S-ID), which includes patients with one or more clinical co-morbidities in addition to ID, and non-syndromic intellectual disability (NS-ID), where ID is the exclusive clinical feature [4]. Distinguishing NS-ID from S-ID is not easy because subtle neurological anomalies and psychiatric disorders are more difficult to diagnose due to the non-specific cognitive impairment [5]. Despite that, many mutations have been identified in genes related to non-syndromic intellectual disability [6,7,8,9]. More than 200 candidate genes have been associated with NS-ID, most of which are not shared between NS-ID and S-ID and/or neurological and neuropsychiatric diseases [10]. Taken all together, these show the efforts being made to understand the etiology of NS-ID and demonstrate the complexity of trying to define this heterogeneous disorder.

## 2. LncRNAs

Long noncoding RNAs form a large and miscellaneous group of non-protein-coding RNAs, defined as transcripts of more than 200 nucleotides in length. They can regulate gene transcription through a great variety of mechanisms, such as: by the interaction with chromatin-modifying complexes, by binding to other RNA-binding factors which can induce or repress transcription, by acting as enhancers, and by acting as decoys [11]. Because of this wide variety of possible functions, they have been identified and described as acting in countless biological processes, including human embryonic development and neurodevelopment.

In the early stages of human fetal growth, it has been shown that lncRNAs represent the most abundant class of transcripts, both during and after embryonic gene activation [12]. Many of them have also been described as human embryonic stem cells (hESCs) pluripotency regulators, like lncPRESS1, that controls a gene network that controls pluripotency [13], and GAS5, a lncRNA that is directly regulated by pluripotency factors and acts on hESCs self-renewal [14]. Additionally, they have also been described during brain development, a very genetically organized and dynamic process [15], where neural induction takes place. Failures in any step of this process can lead to neurological and cognitive impairments [16], highlighting the importance of a fine and coordinated regulation for healthy brain development. Some lncRNAs have already been associated with neural development, such as PNKY, a transcript involved in the suppression of neuronal commitment of neural stem cells (NSCs) [17], FMR4, a lncRNA that promotes the proliferation of human neural precursor cells (NPCs) [18], and splicing variants of the lncRNA C130071C03Riken, which are involved in neural differentiation [19].

In this context, some lncRNAs have also been directly associated to several neurodevelopmental disorders that have intellectual disability as one of their characteristics, like autism spectrum disorder (ASD) [20,21,22,23], Fragile X syndrome [24], Prader-Willi syndrome [25,26,27], Rett syndrome [28], and Angelman syndrome [29,30,31]. However, many neurodevelopmental disorders are associated with defective neural differentiation and circuit formation [32], most genes known to cause these diseases belong to a few molecular pathways that are commonly affected [33]. Additionally, little is known about how lncRNAs may be regulating those pathways and how they can be involved in ID. Indeed, scientific articles regarding “lncRNA and intellectual disability” in the PubMed database returns less than 100 articles spanning the last 27 years, and, in a more stringent search, less than 20 articles if the query is specified for “Text word” or “Title/Abstract”.

In this review, we sought to explore the roles of lncRNAs in the main pathways involved in NS-ID [4,34], in order to contribute to the elucidation of its complex regulation network.

## 3. LncRNAs and Pathways Involved in Non-Syndromic Intellectual Disability and Neural Development

### 3.1. Wnt/β-Catenin Pathway

The Wingless (Wnt) gene family encodes secreted signaling proteins that direct cell proliferation, cell polarity, and cell fate determination during embryonic development and tissue homeostasis [35]. The canonical Wnt signaling pathway (Wnt/β-catenin) governs a developmental cascade by regulating the amount of the transcriptional co-regulator β-catenin within the cell, controlling critical gene expression programs involved in cell proliferation, differentiation, cell adhesion [36], as well as epigenetic/transcriptional regulation [37]. Disbalanced expression of these proteins can disrupt the central nervous system’s normal development and cause loss of neurons, fostering neurological disorders [38,39].

Next-generation sequencing enabled better comprehension of multiple dominant mutations in the gene encoding β-catenin, clarifying its role in cognitive impairment and bringing in vivo evidence that deregulation of this pathway leads to intellectual disability [38,40]. Despite the lncRNAs inability to code for proteins, it is widely accepted that these molecules can modulate a diverse range of biological processes, including the Wnt/β-catenin signaling pathway [41,42].

Notably, the central nervous system expresses a great diversity of lncRNAs, suggesting that their dysregulation plays a critical role in neurological conditions [43,44]. A few lncRNAs have been described to regulate coding genes that are part of the canonical Wnt signaling pathway. For instance, mice experiments showed that downregulation of the Maternally Expressed Gene 3 (MEG3), a lncRNA commonly found overexpressed in neurons of the forebrain of mice [45], modulates the Wnt/β-catenin signaling pathway, enhancing nerve growth and alleviating neurological impairment of rats after brain injury [46]. The lncRNA Neat1 was recently reported to enhance the protein levels of core factors of the Wnt/β-catenin signaling pathway affecting neuroinflammation damage [47]. Further evidence points out that the signaling axis miR-124-Neat1-Wnt/β-catenin plays an essential role in regulating neuronal differentiation, apoptosis, and migration of mouse spinal cord progenitor cells (SC-NPCs) [48]. This study showed that overexpression of miR-124 enhanced the expression of the lncRNA Neat1 and positively regulated mRNAs involved in the Wnt/β-catenin pathway. Although the focus is on the therapeutic effectiveness of miR-124/Neat1 for spinal cord injury, clarifying the role of lncRNAs in promoting neuronal differentiation may be relevant to unravel ID pathways [48].

A modulator of the canonical Wnt/β-catenin signaling pathway, the SOX family of transcription factors, is widely known for its role in the developing nervous system. For instance, Sox2 is expressed in neural stem cells regulating self-renewal and differentiation into neurons [49,50]. However, Sox genes suffer regulation by lncRNAs, as shown by Ng et al., that nuclear lncRNA RMST regulates neuronal differentiation and associates with transcription factor SOX2. The author also reported that Sox2 and RMST regulate common targets and RMST downregulation affects SOX2 genome binding [51]. Other lncRNAs that have also been described interfering in SOX regulation include Sox2ot [52] and lincRNA-RoR [53]. The study of genes that affect neurodevelopment and are associated with diseases that do not start in childhood is also useful to understand intellectual disorders. DISC1 gene, initially described in schizophrenia [54], interacts directly with the GSK3β, inhibiting its activity and leading to β-catenin stabilization. This cascade exerts an influence on neural progenitor proliferation [54]. DISC1 was reported to have the alternative splicing induced by the long non-coding RNA MIAT, leading to its downregulation and disturbing neurodevelopmental mechanisms [55,56].

As shown in this review, much still needs to be unraveled about how long non-coding RNAs regulate genes related to intellectual deficiency. These findings corroborate these molecules’ critical role in controlling different components of the Wnt/B-signaling pathway during the earliest stages of neural development.

### 3.2. Notch Pathway

In the developing nervous system, the Notch signaling pathway is involved in repressing neural differentiation-inducing genes, thus preventing NSCs from differentiating and maintaining these embryonic cells [57,58]. This pathway is important in regulating the balance between stem cell maintenance and proper neuronal differentiation, so the correct timing is achieved, and the necessary quantity of neurons is produced [59]. It is a highly conserved pathway that, in mammals, contains four receptors (Notch 1–4) and five ligands (Dll1, Dll3, Dll4, Jagged-1, and Jagged-2). Upon ligation of the ligand with the receptor, a proteolytic cleavage occurs, releasing the Notch intracellular domain that translocates to the nucleus and promotes transcription of target genes by interacting with the protein RBPJ [60].

It has already been demonstrated that the ablation of the receptor Notch1 in neuroepithelial cells leads to premature differentiation and apoptosis, failing to generate neural cells [61], which may significantly impact the central nervous system (CNS) development. It was also demonstrated that two essential effectors of the Notch signaling are crucial for maintaining the embryonic nervous system structure, keeping the organization and morphology of the cells [62]. Additionally, pathogenic variants of the Notch ligand Dll1 have recently been suggested as causative of a neurodevelopmental phenotype in a cohort whose individuals presented with developmental delay, intellectual disability, and brain malformations [63].

Understanding how lncRNAs may regulate the Notch signaling pathway can bring important highlights about their roles in ID. In 2006, Rani et al. [64] functionally characterized a novel lncRNA, LncND, in a neurodevelopmental disorder related to a microdeletion at 2p25.3, including the LncND locus, and showing ID as one of its phenotypic characteristics. This primate-specific transcript is highly expressed in early neural progenitor cells, and it was demonstrated that it acts as a miRNA sponge. Furthermore, this lncRNA sequesters miR-143-3p and regulates Notch signaling since this miRNA targets NOTCH-1 and NOTCH-2 mRNAs. Through LncND knockdown experiments, the authors showed that cells underwent premature differentiation of neural precursor cells, and through an in vivo overexpression assay, they observed that this lncRNA regulates the expansion of radial glial cells in the murine developing cortex [64].

In an investigation of the transcriptional landscape of embryonic and adult brains, Goff et al. (2015) [65] generated a cohort of 13 lncRNA-null mutant mouse models, which had evidence of expression in neural stem cells and the brain. They observed a more significant number of differentially expressed genes at mouse embryonic day 14.5 (E14.5) compared to adult brains, with significant enrichment of gene sets related to neuronal differentiation and cell fate commitment. In this cohort, one of the mutant strains was a knockout (KO) of KANTR locus. A differential expression analysis between KO and wild-type (WT) for this strain showed a significant increase in gene sets involved in Notch signaling and neural development in the E14.5 brain. Similarly, the deletion of the lncRNA PERIL locus in the E14.5 brain led to a significant increase in the neural stem cell marker genes Notch 1–3, which was also observed with the ablation of the lncRNA PANTR1 locus [65].

The trisomy 21 found in Down Syndrome (DS) is suggested as associated with delayed neuron formation. Recently, it was shown that the lncRNA XIST, a very well-known transcript in the X-chromosome silencing process, is capable of silencing the extra chromosome 21 (chr21) in DS iPSCs. The silencing of the extra chr21 allows a higher rate of neuron production when compared to the non-silenced cells. Through scRNA-seq analysis, it was revealed that the extra chr21 silencing by XIST significantly dysregulated the Notch signaling pathway and also led to differential expression of TTYH1, an enhancer of this pathway. The elevated expression of Notch signaling genes is then suggested to keep NSCs in the proliferative state, delaying their terminal differentiation into neurons [66].

In summary, these studies highlight how lncRNAs may regulate the Notch signaling pathway and play important roles in neuronal differentiation, which is shown to be strongly related to ID development.

### 3.3. Sonic Hedgehog Pathway

The Sonic hedgehog (Shh) pathway plays a role in the development of tissues and organs, including the CNS, and the determination of tissue polarity. Its role in forming the ventral spinal cord model and early embryonic development has been previously observed [67]. Signaling in this pathway is indispensable for a cell’s pattern and fate, especially in the CNS. Throughout vertebrates’ CNS development, Shh is required and acts as a morphogenic factor in proliferation, differentiation, and survival of neural precursor cells [68]. In the brain, Shh protein is first expressed ventrally at the early development stages [69,70].

The Shh pathway is classified as canonical when it involves three main components: begins with the Shh ligand binding to Patched (Ptch) protein receptor, which then interacts with and inhibits smoothened (SMO) protein and ends with the regulation of transcriptional effectors from the Gli family. Non-canonical Shh occurs when the activation of Ptch/SMO independent of Gli or when the transcriptional factor Gli is activated regardless of the Shh ligand of Ptch/SMO [71]. Shh plays an important role in neurogenesis in the adult mammalian brain, while its receptors, Ptch and Smo, are more expressed in the adult hippocampus and progenitors from this region [72].

Studies of gain and loss of function demonstrated that Shh is essential to induce ventral-neural cell types, and its deficiency affects the establishment of spinal cord structures and brain development. In the brain, altered regulations of the Shh signaling can lead to an extensive range of neurological disorders, brain tumors, and intellectual disability [73,74]. Variants in the Shh gene have also been found in patients showing development abnormalities, and speech and learning delays [70,75]. Several non-coding RNAs have been reported as having a role of regulation of the Shh pathway, including lncRNAs, which were shown to function in modulation of neural development and differentiation, with possible effect in the intellectual disability phenotype [76].

The lncRNA AK053922, which is transcribed from the Gli3 locus, is expressed in the early stages of brain development and was shown to have the ability to help specify distinct neuronal cell types by working as a bifunctional transcriptional switch that can either repress or activate Shh signaling [77,78,79]. Also involved in neurogenesis, lncRNA Gm15577 was identified in the Nbs1-deficient mouse model. Yue et al. [80] demonstrated that Gm15577 regulates the RNA expression of Shh, Nerg1, and β-catenin, and has important functions in neuronal growth and neuroplasticity [81].

One thoroughly studied lncRNA that regulates brain development is Evf2 (embryonic ventral forebrain 2), which is transcribed antisense to the Dlx6 gene, and therefore also known as Dlx6as1. The locus from which it is transcribed is a homeobox-containing transcription factor, essential for forebrain neurogenesis [82,83]. Evf2 is upregulated during GABAN neurogenesis [79] and is expressed in the telencephalon’s Shh-responsive cells during embryonic development [82].

Bond et al. [84] used Evf2 loss-of-function mice to study its role in vivo and reported that Evf2 regulates Dlx6 with mediated concentration-dependent transcriptional control by competition, using both trans and cis mechanisms. The paper also demonstrated that Shh signaling in the embryonic ventral forebrain initiates a transcriptional cascade that requires Evf2 and other lncRNAs for proper GABAergic interneuron development. Other authors also found that Shh activates Efv2 in the embryonic forebrain [85] and is expressed at sonic hedgehog-activated interneuron birth sites in mice [86]. Molecular disturbance of the expression of Evf2 in mice caused a reduction in the number of interneurons in the hippocampus. Even though this decrease was resolved in the mature mice, it was perceived as reducing the inhibition of CA1 pyramidal neuron activity, which suggests a functional defect in these cells [84,87].

### 3.4. Growth Factors and Neurotrophic Factors

Growth factors represent a family of extracellular proteins that induce cell growth and support biological settings [88,89]. Various growth factors have been demonstrated to play a role in the regulation of the adult brain development, in highlighting Fibroblast growth factor-2 (FGF-2), Insulin-like growth factor-1 (IGF-1), and Vascular endothelial growth factor (VEGF). These factors bind to a ligand-specific receptor from the tyrosine kinase family, resulting in the autophosphorylation and activation of its receptors, followed by the activation of signaling pathways, such as PI3K/Akt and MAPK/ERK [90]. Several lncRNAs associated with growth factors have been reported as affecting the brain in conditions of oxygen-glucose deprivation and ischemic stroke, namely MALAT1 [91], Neat1 [92], SNHG12 [93], FENDRR [94], and MEG3 [46].

Neurotrophic factors are signaling proteins playing a role during development, and later, in the adult nervous system. Five neurotrophic factors have been identified in mammals, nerve growth factor (NGF), brain-derived neurotrophic factor (BDNF), Glial cell-derived neurotrophic factor (GDNF), neurotrophin 3 (NT-3), and neurotrophin 4/5 (NT-4/5). In this pathway, those proteins interact with tyrosine kinases (Trk) receptors and their co-receptor p75NTR [95]. Neurotrophins play an essential role in the moderation of neuronal survival, growth, and differentiation [96], and the lack of these factors may lead to neurodegeneration [97]. Neurotrophins have been associated with several psychiatric disorders, like depression, bipolar disorder, anxiety, and autism spectrum disorders [98,99,100,101]. Its upregulation is shown to have beneficial effects on a large range of neurological disorders [102]. BDNF, NGF, and NT-3 have also been considered possible etiologies for attention-deficit/hyperactivity disorder [103].

Brain-derived neurotrophic factor (BDNF) is an important protein for neurodevelopment and maintenance and is one of the most abundant growth factors in the human brain [104]. BDNF plays a role in the coordination of neuronal and glial maturation, participating in axonal, dendritic differentiation, and protecting and enhancing neuronal cell survival [105], and is involved in learning and memory processes [106].

The BDNF locus originates the antisense lncRNA named BDNF-AS, which regulates BDNF mRNA and protein levels in cis [107]. Both transcripts are expressed in several tissues, like the brain, muscle, and embryonic tissues, with the BDNF mRNA having 10- to 100-fold higher expression. Inhibition of BDNF-AS caused a 2- to 7-fold increase in BDNF levels, which resulted in elevated neuronal growth, differentiation, survival, and proliferation, both in vitro and in vivo [108,109]. However, the magnitude of the increase of BDNF protein was less than the extent of mRNA upregulation [108]. Thus, BDNF-AS is a promising therapeutic target for neurodegenerative and neurodevelopmental disorders in which BDNF is downregulated [110].

Localized in a novel nuclear compartment enriched in pre-mRNA splicing factors [111], lncRNA MIAT, also known as GOMAFU/Gomafu (in humans) and RNCR2, have been shown to play a role in retinal development, postmitotic neuronal function [112], and brain development [79], however, these functions need further explanation. Overexpression of MIAT was associated with reduced apoptosis of neuronal cells through miR-211 and the neurotrophin GDNF, resulting in relief of hypoxic-ischemic injury in mice [113]. MIAT also plays a role as a regulator of neural and vascular cell function via the MIAT/miR-150-5p/VEGF network, and its knockdown causes cerebral microvascular degeneration, progressive neuronal loss and neurodegeneration, and behavioral deficits in Alzheimer’s disease [114].

MIAT is expressed in a subset of adult mice neurons, including the hippocampus’ CA1 region, which suggests the lncRNA role in neuronal excitatory transmission [112]. In two separate studies, MIAT was found upregulated in the nucleus accumbens of cocaine and heroin users, suggesting an effect of the lncRNA in addictive behaviors [45,115]. The dysregulation of MIAT can lead to neurological disorders and has also been implicated in the pathogenesis of schizophrenia, being downregulated upon neuronal activation [116].

Mercer et al. studied lncRNAs expression in neuronal-glial fate specification and oligodendrocyte (OL) lineage maturation and found that MIAT was differentially expressed, being downregulated in neuronal-OL progenitor, while upregulated during neurogenesis and all stages of oligodendrocytes lineage specification and maturation [79]. Using MIAT lncRNA knockout mice, Ip et al. observed that the animals exhibited no gross development defects. However, results from behavioral tests suggested that the animals displayed a mild hyperactivity phenotype accompanied by an increased level of dopamine in comparison with wild-type controls [117].

### 3.5. Rho Pathway

Small GTPases of the Rho family comprise 20 proteins distributed in subfamilies: Cdc42, Rac, Rho, Rnd, RhoD, RhoBTB, and RhoH [118]. These proteins play a key role in numerous neuronal development processes, mainly in neuronal morphology controlling dynamic events of the actin cytoskeleton as well as dendritic and synaptic plasticity. Therefore, it is not surprising that the dysfunction of Rho GTPases is associated with intellectual disability. Also, several ID-related mutations have been found in genes that encode effectors or regulators of the Rho GTPases: ARHGEF9 (Cdc42 guanine nucleotide exchange factor 9), FGD1 (FYVE, RhoGEF, and PH domain containing 1), OPHN1 (oligophrenin 1), PAK3 (p21 (RAC1)-activated kinase 3), aPIX (Rac/Cdc42 guanine nucleotide exchange factor 6), and TRIO (trio Rho guanine nucleotide exchange factor) [119,120]. However, no data were found in the literature corroborating the association between lncRNAs and the RHO pathway in ID.

### 3.6. MAPK/ERK Pathway

The MAPK/ERK pathway, also known as the Ras-Raf-MEK-ERK, acts on the transduction from extracellular information to the intracellular environment, regulating various intracellular functions, including cell proliferation, differentiation, and survival [121,122,123]. This pathway has been described as acting in the development of the central nervous system, for example, in the embryonic and early postnatal phases, the cascade signals transmitted by MAPK/ERK to progenitor cells inhibit gliogenesis and promote neurogenesis [124]; in neurons, it acts on the dendritic spine stabilization, since it encodes scaffold proteins and adhesion molecules, and participates in the modulation of ionic changes and receptor insertion as well [125].

MAPK/ERK pathway dysfunction has been associated with many neurological pathologies, including ASD [126,127,128]. It was already reported that transient blockade of the MAPK/ERK pathway on postnatal day 6 in mice caused an increase in apoptosis in the forebrain, and in the long term induced autistic behavioral phenotypes, including social deficits, impaired memory, and reduced long-term potentiation (LTP) in adulthood [129].

N-methyl-D-aspartate receptors (NMDARs) are glutamate-gated ion channels widely expressed in the CNS, regulating neuronal communication and synaptic function [130]. Functional NMDARs are heterotetramers formed by two GluN1 subunits and two glutamate binding GluN2 subunits. The GluN2 subunits can be GluN2A–GluN2D, as well as GluN3A and GluN3B, all of which have distinguishing properties and expression patterns in the CNS [131,132]. Several human genetic studies have reported alterations in NMDARs subunits’ genes in a variety of brain diseases, such as intellectual disability, ASD, and epilepsy [133]. Regulation of NMDARs function is a complex process involving numerous proteins in the cell, particularly various protein kinases [134]. The long nucleolus-specific lncRNA (LoNA) has been reported as a regulator of some NMDAR components in mice [135]. LoNA was shown to inhibit rRNA production and ribosome biosynthesis in nucleoli, and eventually, protein synthesis. Additionally, Li et al. showed that levels of synaptic proteins, including NMDA receptor NR1, NR2A, and NR2B, were significantly elevated in the synaptosome fraction isolated from LoNA knockdown mice, ultimately leading to improved neuronal plasticity and long-term potentiation (LTP) [135]. Further studies are required to evaluate whether LoNA may regulate NMDAR components in the context of human neurologic diseases.

SynGAP is a GTPase-activating protein (GAP) selectively expressed in the brain, and that regulates the biochemical signaling in neurons and plays critical roles in neuronal function and brain development [136,137]. This protein is a component of the NMDA-receptor complex and acts downstream of the receptor, blocking the AMPA receptor’s insertion at the postsynaptic membrane by inhibition of the RAS-ERK pathway [138]. SynGAP is a negative regulator of small GTPases, such as Ras and Rap, and is essential for synaptic development, structure, function, and plasticity Mutations in SYNGAP1, which encodes the SynGAP protein, are a major cause of genetically defined childhood brain disorders, and are found in individuals with ID, ASD, severe epilepsy, and schizophrenia [139,140]. Alongside mutations, non-coding RNAs may play a role in the regulation of SynGAP. An antisense lncRNA to SYNGAP1 (SYNGAP1-AS) was upregulated in the ASD postmortem prefrontal cortex and superior temporal gyrus. These findings raised the idea of regulation by the expression of SYNGAP1 mRNA while affecting epigenetic modification of transcription factors and playing a role in ASD pathology and other neurological diseases related to SynGAP deficiency [137,141].

The metastasis-associated lung adenocarcinoma transcript 1 (MALAT1), also known as nuclear-enriched transcript 2 (NEAT2), is a lncRNA consisting of more than 8700 nt located on chromosome 11q13 and discovered as a predictive marker for metastasis in early-stage, non-small cell lung cancer [142,143]. MALAT1 is involved in several biological processes, including cell proliferation, migration, and apoptosis [144,145]. Regarding its role in the nervous system and its related pathologies, MALAT1 was expressed in neurons and induced in response to membrane depolarization [146]. In addition, MALAT1 was described as regulating synapse formation by modulating the expression of genes involved in synapse formation or maintenance [147]. MALAT1 has been related to the pathology of several human neurological diseases, including stroke and Alzheimer’s disease [148]. Concerning the role of MALAT1 in the regulation of the ERK/MAPK pathway, Chen et al. using in vitro differentiation of neuroblastoma-derived Neuro-2a (N2a) cell as a model for the investigation of lncRNAs in neurogenesis, identified that MALAT1 was one of the most significantly upregulated lncRNAs during N2a cell differentiation. The authors also observed that MALAT1 knockdown resulted in defects in neurite outgrowth as well as enhanced cell death and inhibition of the MAPK/ERK signaling pathway. Therefore, it was found to maintain the survival and synaptic formation of neurocytoma cells by activating the ERK/MAPK signaling pathway [149]. In contrast to the finding reported by Chen et al. [149], a recent study conducted by Shi et al. showed the inhibitory effect of lncRNA-MALAT1 on the MAPK/ERK signaling pathway and its influence on neuronal apoptosis in a rat model of cerebral infarction [150].

In addition to MALAT1, other lncRNAs have been described as interacting molecules with the MAPK/ERK signaling pathway in the nervous system’s cells. The long intergenic non-protein-coding RNA p53-induced transcript (LINC-PINT) is a long noncoding RNA induced by p53 and located on the human chromosome 7. In mouse cells, LINC-PINT homologous (LincPint) is involved in Polycomb repressive complex 2 (PRC2) and promotes cell proliferation and survival by regulating the expression of genes of the TGF-b, MAPK, and p53 pathways [151]. Additionally, Blüthgen et al. identified that the transcription of LincPint and other lncRNAs is hampered by MEK inhibition in the murine hippocampus, suggesting that this lncRNA could be a candidate for conveying epigenetic changes initiated by MAPK/ERK [152]. Interestingly, in normal human tissues, LINC-PINT expression presents a positive correlation with members of the MAPK pathway and others [151].

The Nuclear Paraspeckle Assembly Transcript 1 (Neat1) is a ubiquitous, highly expressed, nuclear-retained regulatory lncRNA with essential roles in cellular physiology and pathophysiology [153]. This lncRNA can be processed into two isoforms (NEAT1_1 and NEAT1_2), that accumulate in high levels in the nucleus [154,155] and is involved in carcinogenesis [156] and non-cancerous diseases as well, including neurodegeneration and inflammation [157]. In addition to LincPint, Blüthgen et al. also detected that the two alternatively spliced variants of the lncRNA Neat1 were differentially regulated by MAPK/ERK in their murine model, linking this signaling pathway to the regulation of activity-dependent alternative splicing [152].

### 3.7. Synaptic Vesicle Trafficking and Exocytosis

Defects on the regulation of synaptic vesicle fusion and exocytosis may be associated with neurodevelopmental disorders, since this is a tightly controlled process necessary for neurotransmitters’ release and neuronal communication [158]. Neurotransmitter signaling may influence early developmental events, such as proliferation, migration, and differentiation, once they serve as chemical signals in the nervous tissue [159]. The binding of neurotransmitters to neuron receptors generates electrical signals that alter the neighboring neuron’s morphology and behavior. This process involves gene expression changes that must be maintained for proper maturing neuron development [160].

Alterations that lead to impaired neurotransmission and vesicle trafficking may have a great impact on healthy development. A novel missense mutation identified in the SYN1 gene, for example, was shown to affect synaptic vesicle (SV) clustering at presynaptic terminals of neurons and also spontaneous SV release and mobility, causing synaptic function alterations and inducing an X-linked ID phenotype [8]. A further study showed that the most affected KO mice proteins for the FMR1 gene were associated with signal transduction, neuronal development, and GABA/glutamate neurotransmission. The lack of its encoded protein, FMRP, led to alterations in synaptic vesicles’ unloading dynamics, contributing to the aberrant synaptic transmission in Fragile-X syndrome patients [161].

It has been demonstrated that some lncRNAs could affect intellectual development by modulating synaptic vesicle trafficking and exocytosis. Wang et al. analyzed blood samples of ASD children and compared them with controls. Through differential expression analysis, they demonstrated that the lncRNA SNAP25-AS1, which is derived from the SNAP25 locus, is upregulated and associated with the synaptic vesicle cycling pathway [21]. It is known that the synaptosomal-associated protein 25 (SNAP25), together with syntaxin-1A (STX1A) and vesicle-associated membrane protein 2 (VAMP2), mediates neurotransmitters released by the fusion of synaptic vesicles [162,163]. It was also already reported that alterations in gene expression of SNAP25 in mammals are associated with schizophrenia-like behavior [164], and sequence variations in the SNAP25 locus are associated with attention-deficit/hyperactivity disorder (ADHD) [165,166,167].

It was recently shown that the lncRNA neuroLNC is conserved in mammals and it is tuned by synaptic activity. It was also demonstrated that neuroLNC affects synaptic release due to its interaction with RNA-binding protein TAR DNA binding protein-43 (TDP-43) [168]. This protein colocalizes strongly with endocytic proteins, and it is known to bind several mRNAs that encode synaptic vesicle proteins (e.g., members of syntaxin and synaptotagmin) [169,170]. In addition to TDP-43 participating in diverse RNA processes such as synthesis, splicing, stability, and transport [171,172], its importance is highlighted by the inhibition of the endocytosis process, which may be an underlying cause of disrupted neuronal trafficking causing amyotrophic lateral sclerosis (ALS) [170]. In this sense, it was demonstrated by loss-of-function experiments that TDP-43 might impact signaling and endosomal trafficking in neurons [173].

### 3.8. Transcriptional Regulation and Chromatin Remodeling

Long non-coding RNAs promote transcriptional regulation by different mechanisms [11,174]. A typical function of lncRNAs is to modulate the proteins’ activity by recruiting chromatin-modifying complexes and specifying histone modifications patterns [175]. A recent report demonstrates that the long non-coding FMR4 plays a role as a chromatin-associated transcript with evidence of functioning as a trans-acting lncRNA in neural precursor cells, regulating distant genomic loci [18]. Evidence pointed out that overexpression of FMR4 significantly altered genome-wide histone methylation status regarding H3K4Me3 and H3K27Me3 marks. The FMR4-mediated histone methylation changes affected the expression of neurodevelopmental genes; besides, the authors suggest that the FMR4 putative targets may be related to its function as a positive regulator of neural precursor cell proliferation [18].

The lincRNA Dali study showed the first evidence of an intergenic long noncoding RNA modulating transcriptional programs of genomically distal regulatory elements [176]. Dali controls the expression of a 50 kb upstream transcription factor gene, Pou3f3 (also known as BRN1 or Oct8), which plays a role in developing the nervous system [177]. Dali and Pou3f3 share transcriptional targets, regulating gene expression during neural differentiation. Moreover, Dali directly binds to DNA methyltransferase DNMT1, the BRG1 core component of the SWI/SNF family chromatin remodeling BAF complex, the P66beta, and SIN3A transcriptional co-factors, validating its role in chromatin-modifying proteins regulation [176].

A widely known gene to play a role in gene regulation during neurodevelopment is the MeCP2. The encoded protein can bind DNA and regulate gene expression; moreover, MECP2 mutations and dysfunctions have been associated with intellectual disability in several neurodevelopmental disorders [178,179,180]. The function of MECP2 has been proven broader than a mere transcriptional repressor, as initially believed [179]. MeCP2/lncRNAs-mediated chromatin remodeling received a closer look. Physical association with the lncRNA RNCR3 (retinal non-coding RNA 3) confers new gene regulation mechanisms by affecting chromatin structure [181].

An alternative mechanism used by lncRNAs to perform transcriptional regulation is by targeting splicing factors. The lncRNA Pnky regulates neuronal differentiation of embryonic and postnatal neural stem cells by binding to the polypyrimidine tract-binding protein 1 (PTBP1), a critical splicing factor during neuronal development [17,182]. In NSCs, Pnky binding to PTBP1 regulates neurons’ production by controlling key transcripts related to cell differentiation. The lncRNA Tuna, which also binds to PTBP1, has a role in neurogenesis. However, further clarification is needed if their mechanism is by affecting splicing machinery [183,184].

The lncRNA MIAT is involved in a neurogenic commitment by controlling the differentiation of neural progenitors and newborn neurons’ survival. In vivo experiments showed that Miat overexpression or RNAi silencing altered the splicing pattern of Wnt7b, promoting changes in variants proportion [185]. The lncRNA CAT7 interacts with the Polycomb Repressive Complex (PRC1) by co-immunoprecipitation. The evidence shows that Cat7 cooperates with the PRC1 to promote gene regulation during neuronal differentiation [186].

With respect to lncRNAs binding directly to chromatin to promote transcriptional regulation, experiments of chromatin isolation by RNA precipitation followed by sequencing (ChIRP-seq) showed that the lnc-Nr2f1 binds to chromatin in an isoform-specific form to distinct genomic loci, regulating neuronal genes in mouse, for instance, the gene Nrp2 involved in neural pathfinding [22].

## 4. Concluding Remarks

**Figure 1:**
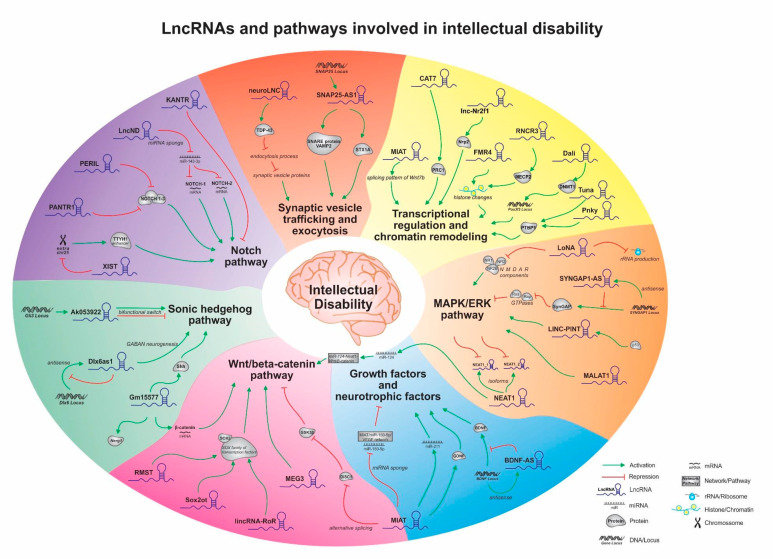
Schematic representation of the mechanisms of action by which lncRNAs may regulate molecular and cellular pathways already associated with non-syndromic intellectual disability and neural function. They play important roles in neurodevelopmental processes, which may have relevant implications for the disease origins and evolution.

LncRNAs have been extensively explored in many biological pathways in recent years and their regulatory importance has been increasingly recognized. Although, their roles in ID are still not deeply understood, leaving a gap in its transcriptional network. This review shows that many lncRNAs are acting in pathways involved in NS-ID (Figure 1). Some transcripts were shown to participate in more than one pathway, reinforcing their versatile mechanisms of action, and some new transcripts were also evidenced. We found a large number of lncRNAs in the transcriptional regulation and chromatin remodeling pathway, followed by Wnt/β-catenin, Notch, and MAPK/ERK pathways (Table 1), possibly representing the main biological pathways in which lncRNAs act during neurodevelopment. On the other side, the remaining pathways might not have been as explored as the first ones, therefore being potential discovery fields. Another point to be considered is that lncRNAs are relatively new in the RNA research field. Consequently, their impact in ID is still quite limited, which is also due to the large amount of data generated by high-throughput experiments together with their lack of functional characterization. In this sense, we consider this a quite intriguing and fast-growing research area that will be better understood in the future, as the number of researchers in this field increases, and new experimental tools and approaches are developed to explore the diverse roles that lncRNAs play in ID.

Altogether, these evidences emphasize the relevance of lncRNAs in pathways involved in NS-ID, which strongly indicates their relevance in this disorder. Additionally, these studies expand the knowledge about lncRNAs regulatory roles during neurodevelopmental events and demonstrate the highly complex gene networks involved in this process, making it necessary to assess further and validate their NS-ID roles.

## Figures and Tables

**Table 1:** Overview of lncRNAs roles in each non-syndromic intellectual disability-related pathway.

Pathway	LncRNA	Ensembl Gene ID	Biotype ^1^	Role in the Pathway	Reference
Wnt/β-catenin pathway	*MEG3*	ENSG00000214548	Intergenic	It modulates the Wnt/β-catenin signaling pathway, enhancing nerve growth and alleviating neurological impairment of rats after brain injury.	[46]
	*Neat1*	ENSG00000245532	Intergenic	The signaling axis miR-124-Neat1-Wnt/β-catenin plays an important role in regulating neuronal differentiation, apoptosis, and migration of mouse spinal cord progenitor cells.	[48]
	*RMST*	ENSG00000255794	Intergenic	Nuclear lncRNA *RMST* regulates neuronal differentiation and associates with transcription factor SOX2.	[51]
	*Sox2ot*	ENSG00000242808	Sense overlapping	Its expression is inversely correlated to Sox2 expression during neural differentiation of mouse ESCs.	[52]
	*lincRNA-RoR*	ENSG00000258609	Intergenic	*lincRNA-RoR* participates in a regulatory loop, together with Sox2, to help maintain hESC self-renewal balance and may contribute to genetic networks’ regulation during development.	[53]
	*Gomafu*	ENSG00000225783	Intergenic	The long non-coding RNA *Gomafu* induces alternative splicing of DISC1, leading to its downregulation and disturbing neurodevelopmental mechanisms.	[45,56]
Notch	*LncND*	NA	Intergenic	It sequesters miR-143-3p, which targets NOTCH1 and NOTCH2 mRNAs. Knockdown of this lncRNA led to premature precursor cells’ differentiation in humans and its overexpression regulates radial glial cells’ expansion in murine developing cortex.	[64]
	*KANTR*	ENSG00000232593	Sense overlapping/Sense intronic	Knockout mice of *KANTR* locus increased gene sets involved in Notch signaling and neural development.	[65]
	*PERIL*	NA	NA	Knockout mice of these lncRNAs locus increased the neural stem cell marker genes NOTCH1–3.	[65]
	*PANTR1*	ENSG00000233639	Intergenic		
	*XIST*	ENSG00000229807	Intergenic	*XIST* silencing of the extra chr21 in Down Syndrome hiPSCs led to diminished Notch pathway signaling and a higher rate of neuron production.	[66]
Sonic hedgehog	*AK053922*	NA	NA	It helps to specify distinct neuronal cell types through acting as a bifunctional transcriptional switch that can either repress or activate sonic hedgehog (Shh) signaling.	[77,78]
	*Gm15577*	ENSMUSG00000086708	Antisense	In mice, it modulates *Shh* mRNA expression, playing important roles in neuronal growth and neuroplasticity.	[80]
	*Evf2/DLX6-AS1*	ENSG00000231764	Antisense	*Evf2* is required for proper GABAergic interneuron development, through a transcriptional cascade initiated by Shh signaling in the embryonic ventral forebrain.	[82,86]
Growth and neurotrophic factors	*BDNF-AS*	ENSG00000245573	Antisense	Regulates BDNF mRNA and protein levels, which are critical for the development, survival, and maintenance of neurons in the nervous system.	[109,110]
	*MIAT/Gomafu*	ENSG00000225783	Intergenic	It is involved in brain development and regulation of neural and vascular cell function via the *Gomafu*/miR-150-5p/VEGF network.	[111,112]
MAPK/ERK	*LoNA*	NA	NA	Knockdown of *LoNA* led to an increase of NR1, NR2A, and NR2B proteins in mice and was found in association with improved neuronal plasticity and long-term potentiation.	[135]
	*SYNGAP1-AS1*	ENSG00000274259	Antisense	*SYNGAP1-AS* is supposed to regulate the expression of *SYNGAP1* mRNA in the prefrontal cortex and superior temporal gyrus of patients with autism spectrum disorders.	[137]
	*MALAT1*	ENSG00000251562	Intergenic	Knockdown of *MALAT1* resulted in the inhibition of the MAPK/ERK pathway in mouse N2a cells and also could inhibit this signaling pathway in a rat model of cerebral infarction.	[149,150]
	*LINC-PINT*	ENSG00000231721	Intergenic	In mice, its homologous (LincPint) regulates genes of the MAPK pathway and its transcription is hampered by MEK inhibition in the murine hippocampus. In human normal tissues, the expression of *LINC-PINT* was positively correlated with the expression of the MAPK pathway genes.	[151,152]
	*Neat1*	ENSG00000245532	Intergenic	This transcript is processed into two isoforms that are involved in the pathogenesis of human neurodegenerative diseases and, in mice, its alternatively spliced variants are differentially regulated by the MAPK/ERK pathway.	[157]
Synaptic vesicle trafficking and exocytosis	*SNAP25-AS1*	ENSG00000227906	Antisense	In ASD patients, it is upregulated and associated with the synaptic vesicle cycling pathway.	[21]
	*NeuroLNC*	NA	NA	It interacts with TDP-43, affecting synaptic vesicle release, which may be the cause of disrupted neuro-trafficking in amyotrophic lateral sclerosis.	[168]
Transcriptional regulation and chromatin remodeling	*FMR4*	ENSG00000268066	Antisense	The *FMR4*-mediated histone changes affected the expression of neurodevelopmental genes and its targets may be related to its function as a positive regulator of neural precursor cell proliferation.	[18]
	*Dali*	NA	Intergenic	*Dali* controls the expression of the transcription factor gene Pou3f3 (also known as BRN1 or Oct8), which in turn plays a role in the development of the nervous system.	[177]
	*RNCR3*	ENSG00000253230	Intergenic	Physical association of MECP2 with the lncRNA *RNCR3* confers new mechanisms of gene regulation by affecting chromatin structure.	[181]
	*Pnky*	ENSMUSG00000107859	NA	It regulates neuronal differentiation of embryonic and postnatal neural stem cells by binding to the PTBP1 protein.	[17,182]
	*Tuna*	ENSG00000250366	Intronic	The lncRNA *Tuna* binds to PTBP1, with a possible role in neurogenesis. However, the mechanism needs to be further investigated.	[183]
	*MIAT*	ENSG00000225783	Intergenic	It is involved in a neurogenic commitment by controlling the differentiation of neural progenitors and the survival of newborn neurons.	[185]
	*CAT7*	NA	NA	*CAT7* cooperates with PRC1 to promote gene regulation during neuronal differentiation.	[186]
	*lnc-Nr2f1*	ENSG00000248588	Antisense	*lnc-Nr2f1* binds to chromatin in an isoform-specific way to distinct genomic loci, regulating neuronal genes in mice.	[22]

^1^ Biotypes were obtained with the LNCipedia database (https://lncipedia.org/, accessed on 11 November 2020). NA: Not available.
